# *Kjellmaniella crassifolia* Reduces Lipopolysaccharide-Induced Inflammation in Caco-2 Cells and Ameliorates Loperamide-Induced Constipation in Mice

**DOI:** 10.4014/jmb.2407.07036

**Published:** 2024-10-01

**Authors:** Kirinde Gedara Isuru Sandanuwan Kirindage, Arachchige Maheshika Kumari Jayasinghe, Mi-Soon Jang, Ka-Jung Lee, Hyun-Jung Yun, Ginnae Ahn, Jae-Young Oh

**Affiliations:** 1 Department of Food Technology and Nutrition, Chonnam National University, Yeosu 59626, Republic of Korea; 2 Food Safety and Processing Research Division, National Institute of Fisheries Science, Busan 46083, Republic of Korea

**Keywords:** Inflammation, tight junctions, intestinal barrier, inflammatory cytokines, NF-κB, constipation

## Abstract

Gastrointestinal disorders are widespread globally, with inflammatory diseases being particularly prominent. This study aimed to investigate the effect of *Kjellmaniella crassifolia* hot water extract (KCH) on lipopolysaccharide (LPS)-induced inflammation in human intestinal epithelial (Caco-2) cells and loperamide-induced constipation in BALB/c mice. The study’s findings revealed that KCH dose-dependently increased the cell viability and reduced the NO production by decreasing the iNOS and COX-2 expression in LPS-stimulated Caco-2 cells. Also, KCH downregulated the mRNA expression of pro-inflammatory cytokines (IL-1β, IL-6, IL-8, and TNF-α) by regulating the activation of MAPK and NF-κB signaling pathways in LPS-stimulated Caco-2 cells. In addition, KCH increased the expression levels of tight junction proteins, occludin, ZO-1, and claudin-1 in a dose-dependent manner. Furthermore, in vivo study outcomes demonstrated that KCH improved intestinal transit, increased fecal moisture content, and reduced fecal impaction in constipated mice. KCH decreased the mRNA expression of pro-inflammatory cytokines (IL-1β, IL-6, IL-8, and TNF-α), thereby increasing the expression levels of intestinal tight junction proteins (occludin, ZO-1, and claudin-1) in the small intestine tissues of the experimental mice. These proteins may help regulate intestinal motility and improve stool passage, thus reducing constipation. These findings suggest that KCH could be a promising functional food ingredient for managing intestinal inflammation, inflammation-related disorders, constipation, and the pathophysiology of constipation.

## Introduction

Inflammatory bowel disease (IBD) is a condition characterized by chronic inflammation of the digestive tract [1]. Constipation is identified as a common symptom of IBD and reportedly, over 10% of IBD patients have chronic constipation, diagnosed by difficulty in bowel movements [2]. Apart from that, constipation is a common gastrointestinal condition associated with several disorders or medications [3]. This ailment is marked by infrequent bowel movements along with difficulty passing stools and a sensation of incomplete evacuation [4]. Persistent constipation may result in complications such as hemorrhoids, anal fissures, and fecal impaction [4]. Additionally, the physical discomfort associated with constipation increases psychological stress and anxiety.

Moreover, constipation induces several pathological changes within the gastrointestinal tract that initiate a cascade of adverse effects. Prolonged stool retention in the colon increases water absorption which causes harder/drier stools and makes it more difficult to pass. The mechanical strain in harder stool passes can cause micro-tears in the intestinal mucosa which promotes localized inflammation and disrupts the mucosal barrier integrity [5, 6]. Constipation facilitates the overgrowth of pathogenic bacteria by altering the gut microbiota composition [7]. This dysbiosis fosters the production of harmful metabolites, including lipopolysaccharides (LPS), which are endotoxins present in the outer membrane of Gram-negative bacteria [8]. Elevated levels of LPS in the gut can penetrate the compromised mucosal barrier, entering the bloodstream and eliciting systemic inflammatory responses [9].

Finding sustainable and multifaceted treatments for gastrointestinal dysfunctions is vital because of the complex interplay between IBD, constipation, and post-constipation inflammatory conditions. Seaweed materials have demonstrated promise in treating constipation and its associated inflammatory conditions [10, 11]. Additionally, the anti-inflammatory and antioxidative properties of seaweed components can help mitigate lipopolysaccharide-induced toxicity and support mucosal healing [12]. The importance of natural treatments lies in their potential to offer a sustainable and integrated approach, addressing both the symptoms and underlying causes without side effects commonly associated with conventional pharmaceuticals.

A common group of marine macroalgae known as kelp has a few prominent species including *Nereocystis luetkeana*, *Macrocystis pyrifera*, and *Saccharina japonica*. *Kjellmaniella crassifolia* also comes under the kelp which is a brown macroalga that has been traditionally used in various cultures for its medicinal properties, particularly in treating constipation [13]. Kelp has been extensively studied for its potential in a range of aspects as a functional food ingredient. Among them, many studies have reported the gastrointestinal tract-related protective effects and immunomodulatory effects of various polysaccharide extracts of *K. crassifolia* [14-16]. Yue Hu and the team have found the gastric protective activities of fucoidan from *K. crassifolia* through the NF-κB signaling pathway [15]. Huimin and the team reported that *K. crassifolia* extract containing 23% alginate, 4.5% laminarin, 5% fucoidan, and 67.5% other compounds, including other types of β-glucan modulates intestinal and systemic immune responses in bagg albino (BAlb/c) mice [17]. Moreover, studies have shown the prebiotic properties of kelp [18]. It can positively influence gut microbiota, fostering a more balanced microbial environment that can further prevent constipation and its related complications. Based on the present status of the understanding of the effects of kelp on gut health, the study was conducted by hypothesizing hot water extract of *K. crassifolia* (KCH) abate LPS-induced inflammatory reactions in colon carcinoma-derived Caco-2 cells since it has been widely used as a model of the intestinal epithelial barrier. Moreover, the study examined the effects of KCH on loperamide-induced constipation in BAlb/c mice to evaluate the multifaceted effects of KCH against IBD symptoms in vivo.

## Materials and Methods

### Materials

Dulbecco’s modified eagle medium (DMEM), and a mixture of streptomycin and penicillin (P/S) were purchased from GibcoBRL (USA). Fetal bovine serum (FBS) was purchased from Welgene (Republic of Korea). 3-(4,5-dimethylthiazol-2-yl)-2,5-diphenyltetrazolium bromide (MTT), Dimethyl sulfoxide (DMSO), bovine serum albumin (BSA), Loperamide hydrochloride, Carmine, Folin and Ciocalteu’s phenol reagent, and gallic acid, were bought from Sigma-Aldrich (USA). D-glucose was purchased from Junsei Chemical Co., Ltd. (Japan). The stool softener was purchased from Dulcolax, Dulcolax (USA). BCA protein assay kit, NE-PER nuclear and cytoplasmic extraction kit, 1-Step transfer buffer, Pierce RIPA buffer, protein ladder, and SuperSignal West Femto Maximum Sensitivity Substrate were purchased from Thermo Fisher Scientific (USA). Antibodies for the western blot analysis were purchased from Santa Cruz Biotechnology Inc., (USA) and Cell Signaling Technology Inc.(USA). Skim milk powder was obtained from BD Difco (Sparks, USA). Normal goat serum, Prolong Gold antifade reagent with DAPI reagent, and DyLihgt 554 Phalloidin were purchased from Cell Signaling Technology. The remaining chemicals and reagents used were of analytical grade.

### Sample Collection, Identification and Extraction

*K. crassifolia* was collected from the south coast of the Korean peninsula. The hot water extraction was done by following the method outlined in the previous study with minor adaptations [19]. In brief, air-dried *K. crassifolia* was pulverized and, 50 g of powder was extracted into 1 L of distilled water at 95°C for 4 h. Then, the extract was filtered through a vacuum filtration apparatus equipped with Whatman grade 42 ashless filter paper which has a 2.5 μm pore size (Particle retention). The filtrate was frozen at −80°C for 4 h, freeze-dried, and KCH was kept in an air-tight container at −20°C for further analysis and experiments.

### Compositional Analysis of KCH

The total phenol content, total protein content, and total carbohydrate content of KCH were evaluated as described in a previous publication [20]. Briefly, KCH was mixed with deionized water and the total phenol content was evaluated by measuring absorbance values at 725 nm in the SpectraMax M2 microplate reader (Molecular Devices, USA). The Lowrys’ protein measuring method was used to evaluate the total protein content in KCH. The absorbance values were measured at 750 nm in the microplate reader. The KCH sample was mixed with phenol and sulfuric acid to determine the total carbohydrate content. After following the procedure, the absorbances of the resultant mixtures were measured at 480 nm. A series of gallic acid, bovine serum albumin, and D-glucose concentrations were used as standards to develop standard curves to evaluate the phenol content, total protein content, and total carbohydrate content in KCH. Sulfated polysaccharides from KCH were isolated by implementing the partially modified method described in one of the previous research [21]. In brief, alginate was collected as calcium alginate and the ethanol was gradually mixed with KCH while stirring and kept for 24 h at 4°C. The mixture was centrifuged to obtain the pellet. The pellet was dissolved in distilled water and then freeze-dried to obtain polysaccharide powder.

### Cell Culture

Mycoplasma negative Caco-2 cells were cultured in DMEM media supplemented with 10% heat-inactivated FBS and 1% penicillin/streptomycin mixture. Cells were maintained under a controlled humidified environment at 37°C with 5% CO_2_ and sub-cultured once in 2 days until exponential growth was preferable for seeding. Cells at their exponential growth were used for further experiments.

### Cytotoxicity of KCH In Vitro

Caco-2 cells were seeded at 1 × 10^4^ cells/well in a 96-well plate. After 24 h of incubation, cells were treated with KCH sample concentrations series (31.3, 62.5, and 125 μg/ml) and incubated for 2 h. Then, incubated alone for another 24 h or stimulated with LPS and incubated for 24 h. Following that, cell viabilities were analyzed using the MTT assay following a pre-described method by Kirindage *et al*. [22].

### Evaluation of Nitric Oxide (NO) Production In Vitro

The effect of the KCH sample on LPS-induced NO production in Caco-2 cells was analyzed by following the method outlined in a previous study with few changes [23]. Concisely, The Griess assay was implemented to measure NO production in LPS-treated Caco-2 cells. The procedure described under the subtopic 'Cell Culture' was followed up to 24 h of incubation after sample treatment and stimulation. Then cell-cultured media was transferred to a labeled 96-well plate. A Griess reagent (1% sulfanilamide and 0.1% N-[naphthyl] ethylenediamine dihydrochloride in 2.5% H_3_PO_4_) was mixed with a cell-cultured media from each well. The absorbance at 540 nm was measured after keeping it at room temperature in the dark for 10 min by using the microplate reader.

### Western Blot Analysis

Western blot analysis was conducted according to the pre-described method with minor changes [24]. Briefly, Caco-2 cells were seeded in 6 cm cell culture dishes. The sample treatment, stimulation, and incubation were done according to the method described in “section 2.6”. Then, cells were harvested by trypsinization and lysed by using either NE-PER nuclear and cytoplasmic extraction kit or RIPA buffer after incubation for either 30 min or 24 h accordingly. After that, standardized protein resolves, band development, and visualization was done by following the method outlined in a previous study [25].

### Quantitative Reverse-Transcription- (qRT-) PCR Assay

After cell culture, sample treatment, and stimulation according to the method in “section 2.6.” RNA isolation and cDNA synthesis were done by following the method in a previous study [26]. Synthesized cDNAs were reacted with PowerSYBR Green PCR Master Mix (Life Technologies LTD, UK) to amplify the corresponding genes in QuantStudio 3 Real-Time PCR System (Life Technologies, USA). The 2^-ΔΔCt^ method was employed to quantify gene expression levels using RT-PCR. Cycle threshold (Ct) values for the target gene and the reference gene were first recorded. ΔCt was calculated for each sample by subtracting the Ct value of the reference gene from that of the target gene. To determine ΔΔCt, the ΔCt of the control sample was subtracted from the ΔCt of the experimental sample. The fold-change in gene expression was then calculated using the formula 2^-ΔΔCt^, which provides a comparative measure of the target gene expression in experimental versus control conditions. The sequences of primers used in the study are listed in one of the prior studies [26].

### Immunocytochemical Analysis

Immunocytochemical analysis was done to evaluate the nuclear translocation of NF-κB p65 in Caco-2 cells. The following method was described in a previous study [27]. In brief, Caco-2 cells were cultured in chamber slides. The sample treatments and stimulation were carried out according to the method mentioned in “section 2.9”. After 30 min of stimulation, wells were rinsed with PBS, fixed, and incubated with a blocking buffer for 1 h before being incubated overnight with anti-NF-κB p65 primary antibodies. Following that, cells were again rinsed with PBS and treated with Alexa Fluor 488 conjugated Anti-Mouse IgG for 2 h. Then, the slide was washed with PBS and covered with coverslips with Prolong Gold antifade reagent containing DAPI. The fluorescent images were captured by using EVOS M5000 (Thermo Fisher Scientific) Imaging microscope.

### Mice Preparation and Experimental Design

**Fig. 1:**
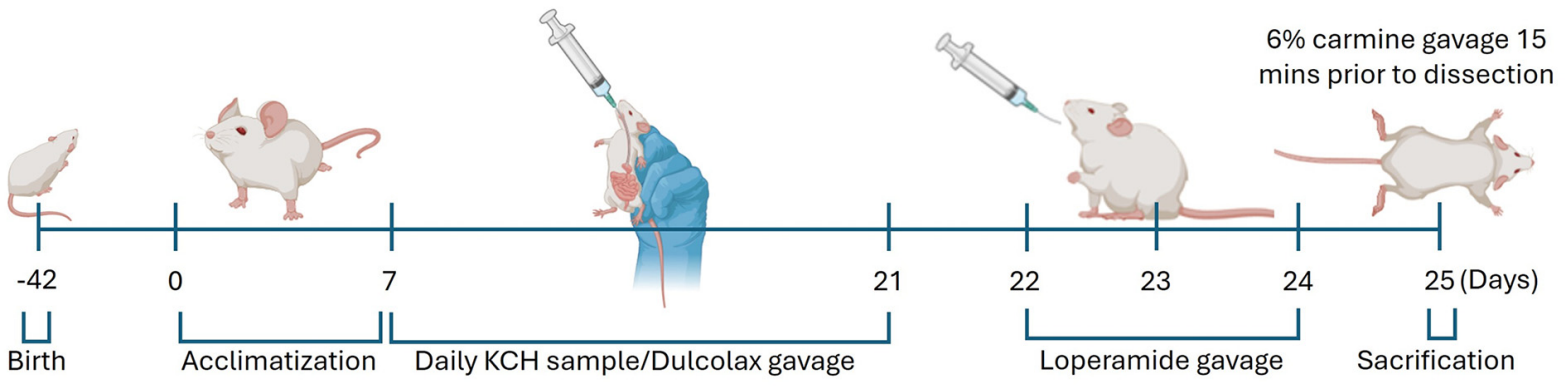
Schematic representation of the in vivo experimental actions with key time points indicated.

Six-week-old female BAlb/c mice which had 18±2g body weight were purchased from Orient Bio, Inc.(Republic of Korea) and were housed in a 12 h day/night cycle environment under 23 ± 2°C temperature. Relative humidity in the housed facility was maintained at 50 ± 10% throughout the experimental period with food and water ad libitum. The timeline of in vivo experiments is indicated in Fig. 1. After 7 days of acclimatization in the housing facility, mice were randomly divided into 5 experimental groups, and oral administration of sample /Dulcolax (positive control) was done for adjacent 14 days. Then the mice were rested for 1 day. Loperamide was orally administrated in the next 2 days to induce experimental constipation. The grouping of mice as well as sample treatments are mentioned in Table 1. Ethical approval was granted to use BAlb/c female mice in the in vivo study by the ethical committee of Chonnam National University, Republic of Korea under permission number CNU IACUC-YS-2022-4.

### Evaluation of Body Weight Variation, Fecal Water Content, and Fecal Impaction

The body weight of each mouse was measured every morning before oral administration of treatments for 18 days starting at the end of the acclimatization period and the average body weight of each group was plotted every day. The feces were collected in the morning for the same days before the oral administration of treatments. Fecal water content was measured by weighing the feces and dried at 100°C for 24 h. The fecal impact of each mouse at the end of the experimental period was evaluated by counting the number of hardened fecal pellets observed in the large intestine and the rectum after dissection.

### Statistical Analysis

IBM SPSS software (Version 24.0, USA) was used for each statistical analysis of this study. Replicates were used in the experiments while using mean ± standard error (SE) to present all experiment data. One-way analysis of variance (ANOVA) along with Duncan’s multiple range test was performed to evaluate the significant variations between data and values at *p* < 0.05 were considered statistically significant.

## Results

### Extraction Yield and Proximate Composition of KCH

According to the findings indicated in Table 2, the polysaccharide content was relatively higher than the polyphenol composition and protein content of KCH on a dry basis (%). Proteins in the KCH were determined as the second highest portion and the total polyphenols were the lowest composition. According to the analysis, 27.82 ± 0.82% of the yield was identified as sulfated polysaccharides, while 11.26 ± 0.19% was identified as alginate. Prominent monosaccharides were identified as Fucose, Rhamnose, Galactose, and Glucose in the sample (Figs. S1 and S2).

### Cytoprotective Effects of KCH on Caco-2 Cells

**Fig. 2:**
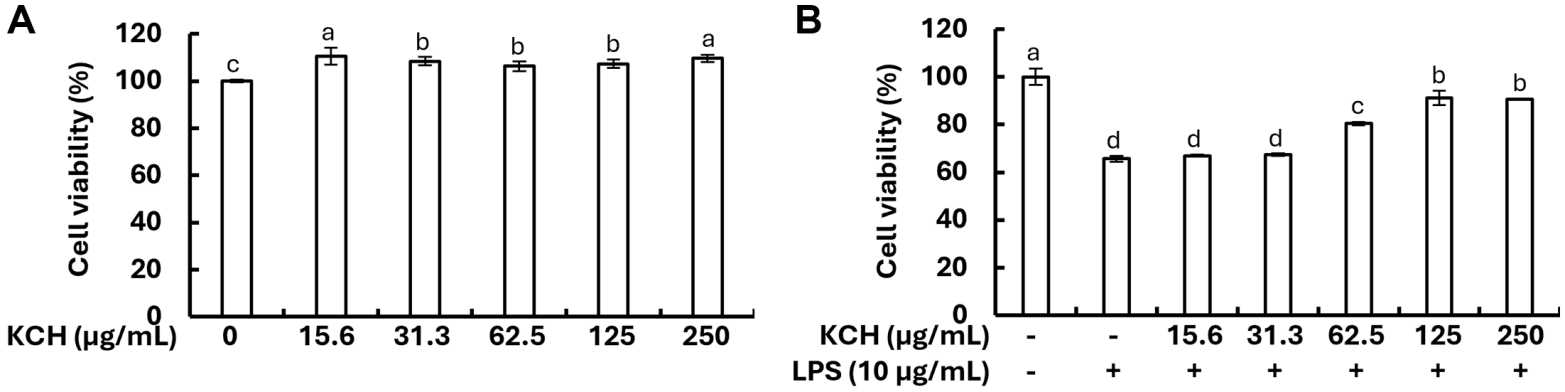
Effect of KCH on (A) the cytotoxicity of Caco-2 cells, and (B) the cell viability (%) in LPS-stimulated Caco-2 cells. All experiments were performed in triplicate (*n* = 3) to ensure reproducibility. Significantly different results are indicated by distinct letters on the error bars (*p* < 0.05).

The concentrations of KCH used in the cytotoxicity analysis were not cytotoxic on the Caco-2 cells up to 250 μg/ml (Fig. 2A). Cell viability of LPS-treated Caco-2 cells was decreased compared to control cells, while KCH treatment significantly increased the cell viability in higher concentrations (Fig. 2B). Based on the findings, concentrations of 31.3 μg/ml, 62.5 μg/ml, and 125 μg/ml were used throughout the study in vitro experiments.

### Anti-Inflammatory Effect of KCH in LPS-Stimulated Caco-2 Cells

**Fig. 3:**
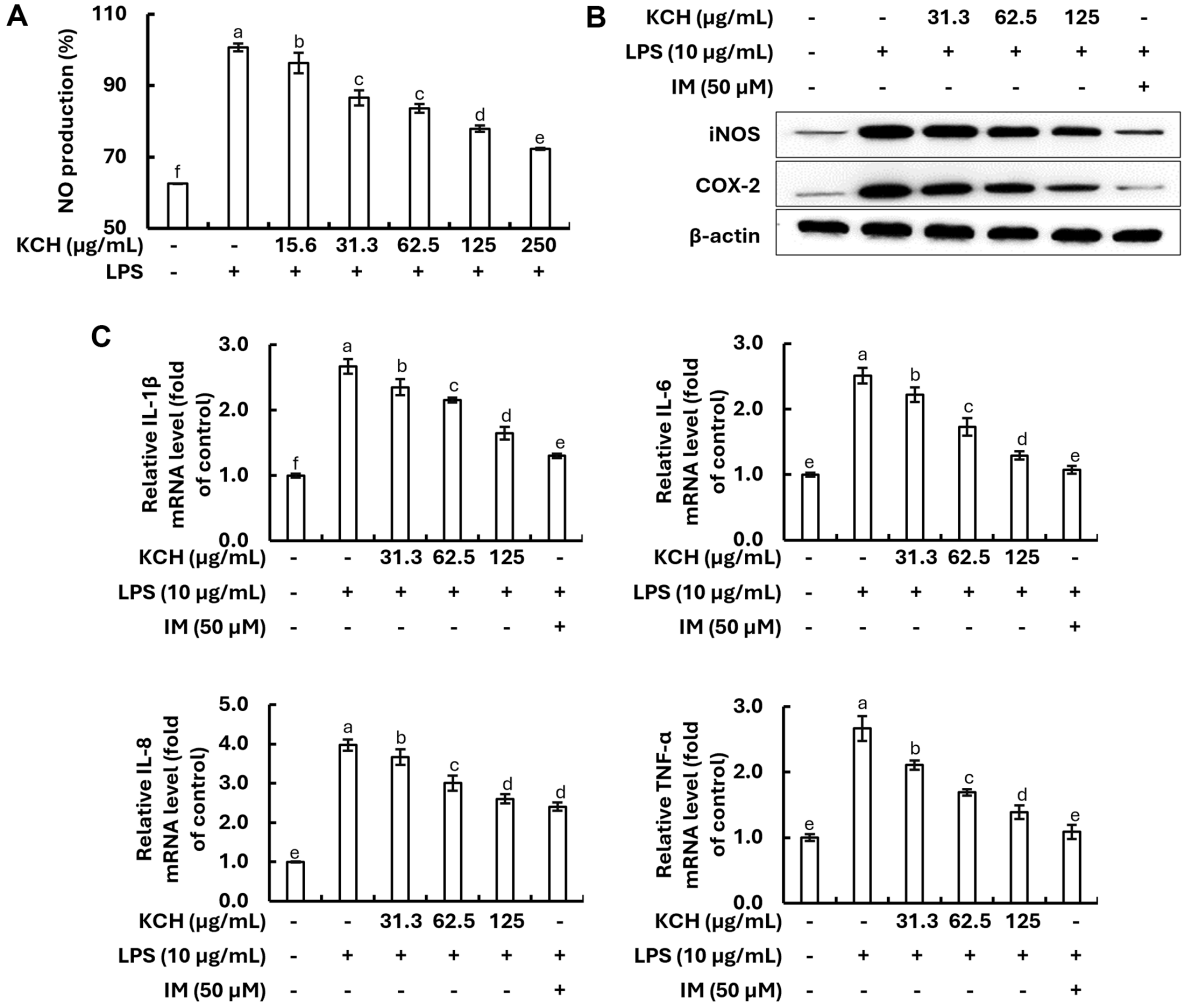
Effect of KCH on (A) the NO production, (B) the protein expression levels of iNOS/COX-2, and (C) the mRNA expression of inflammatory cytokines in LPS-stimulated Caco-2 cells. Data represents results from three independent experiments (*n* = 3) and are expressed as mean ± standard error (SE). Different lowercase letters indicated significantly different results (*p* < 0.05).

**Effect on NO production, and iNOS/COX-2 and mRNA expression of inflammatory cytokines.** The effect of KCH on NO production in LPS-stimulated Caco-2 cells was investigated using the Griess assay. NO production due to LPS stimulation was significantly suppressed by KCH treatment (Fig. 3A). Subsequently, the inhibitory effect of KCH on iNOS and COX-2 expression was examined, and the protein expression was enhanced by LPS stimulation, whereas KCH dose-dependently downregulated (Figs. 3B and S3). Furthermore, qRT-PCR analysis indicated the markedly increased mRNA expression levels of IL-1β, IL-6, IL-8, and TNF-α following LPS stimulation, while pretreatment with KCH considerably attenuated it in a dose-dependent manner (Fig. 3C).

**Fig. 4:**
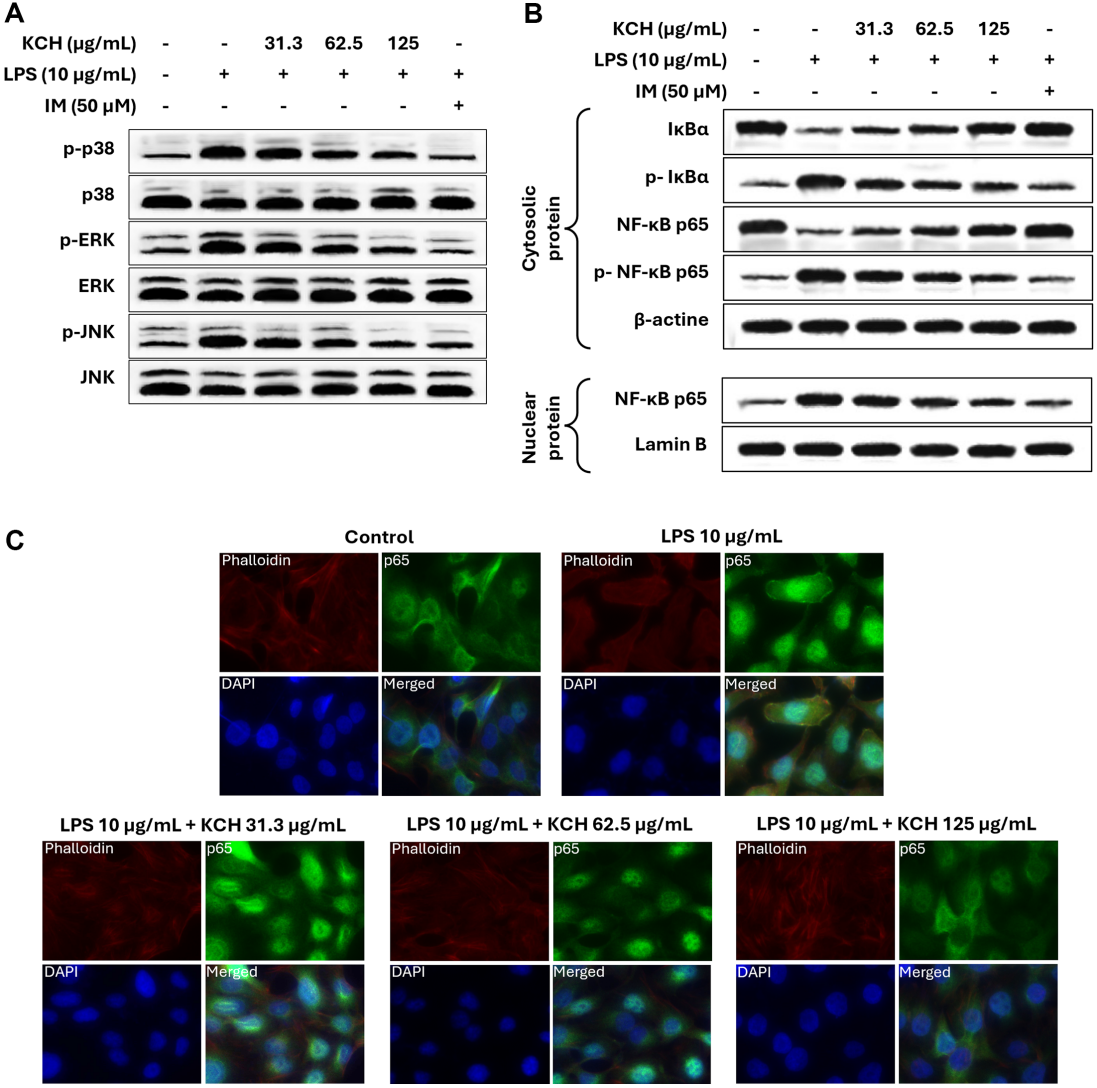
Effect of KCH on the protein expression levels of (A) MAPK, and (B) NF-κB signaling pathways, and (C) the immunofluorescence analysis of NF-κB p65 nuclear translocation in LPS-stimulated Caco-2 cells.

**Effect on MAPK and NF-κB signaling pathways.** The phosphorylation of MAPK signaling molecules, including p38, ERK, and JNK, was remarkably augmented in LPS-stimulated Caco-2 cells, while dose-dependently decreased by KCH (Figs. 4A and S4A). Also, activation of NF-κB signaling is triggered by LPS stimulation, leading to phosphorylation and allowing translocation of NF-κB p65 into the nucleus. Figs. 4B, S4B, and S4C showed that phosphorylation of cytosolic IκBα and NF-κB p65 increased by LPS stimulation and significantly decreased by KCH in a dose-dependent manner. Similarly, KCH downregulated the nuclear translocation of NF-κB p65 in LPS-stimulated Caco-2 cells. Moreover, nuclear translocation of NF-κB p65 was assessed by immunofluorescence analysis. The outcomes further revealed that LPS stimulation increases NF-κB p65 nuclear translocation compared to the control group while dose-dependently decreasing with KCH (Fig. 4C).

**Fig. 5:**
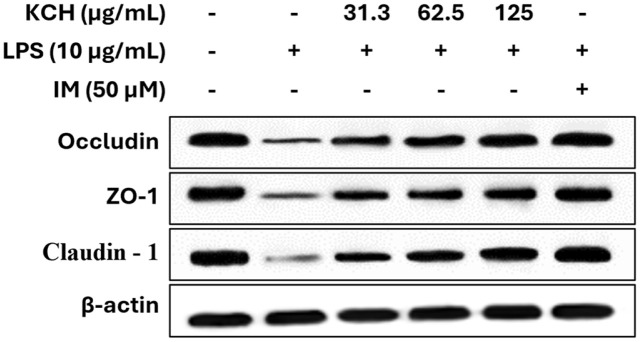
Effect of KCH on the intestinal barrier-related protein expression levels in LPS-stimulated Caco-2 cells.

**Effect on barrier functions.** The effect of KCH on protein expressions related to the intestinal barrier molecules was evaluated using western blot analysis. The protein expression of intestinal barrier-related molecules, including occludin, ZO-1, and claudin-1 was significantly upregulated by the KCH in a dose-dependent manner (Figs. 5 and S5).

### Effect of KCE Treatment against Loperamide-Induced Constipation in BAlb/c Mice

**Fig. 6:**
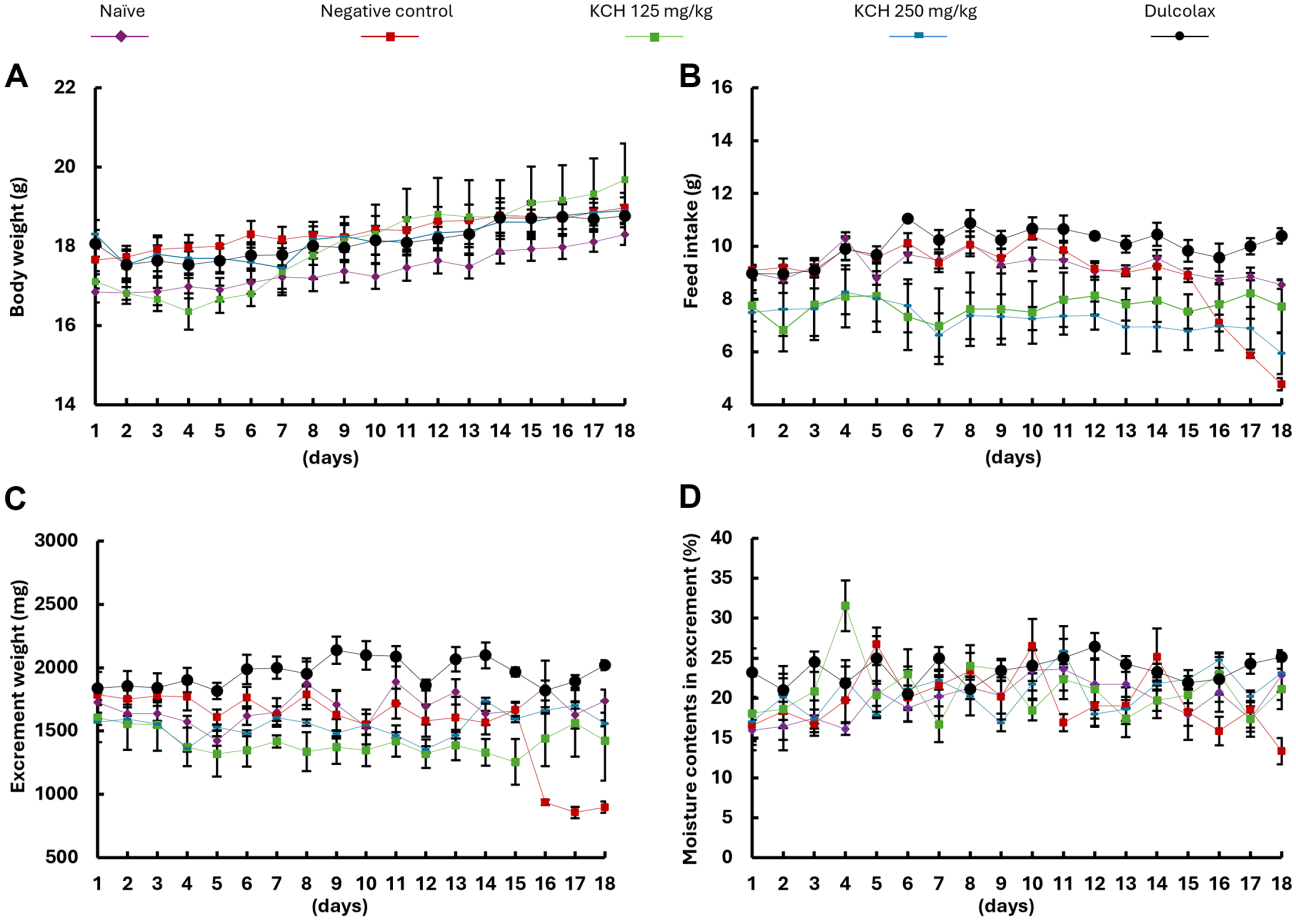
Effect of KCH on (A) the body weight, (B) the feed intake, (C) the excrement weight, and (D) the moisture content in loperamide-induced constipated mice. Results are expressed as the means ± SE of the mean and the experimental model used four replicates (*n* = 7 or 8) to confirm reproducibility.

**Effect on food intake and feces parameters.** Fecal parameters are considered as indications of the induction of constipation in animals [28]. The dry weight of the fecal maters and the moisture content were measured as feces parameters in this study. In addition, changes in the body weight and average feed intake were measured due to a tight relationship with constipation conditions. Average changes in body weight indicated gradual increments between the first and the last day of the experiment period. The body weight changes in adjacent days in each experimental group were indicated in Fig. 6A. Loperamide significantly reduced the food intake (Fig. 6B). In contrast to the loperamide-induced constipation group, KCH gavage increased the food intake in a dose-dependent manner. Parallel to the feed intakes, fluctuation of the excrement weight indicated the same pattern which is indicated in Fig. 6C. Interestingly, the moisture content percentage in fecal pellets from the loperamide-induced constipation group was significantly lower than the KCH sample treatments and positive control (Dulcolax) group. KCH treatment increased the moisture content in fecal pellets dose-dependently (Fig. 6D).

**Fig. 7:**
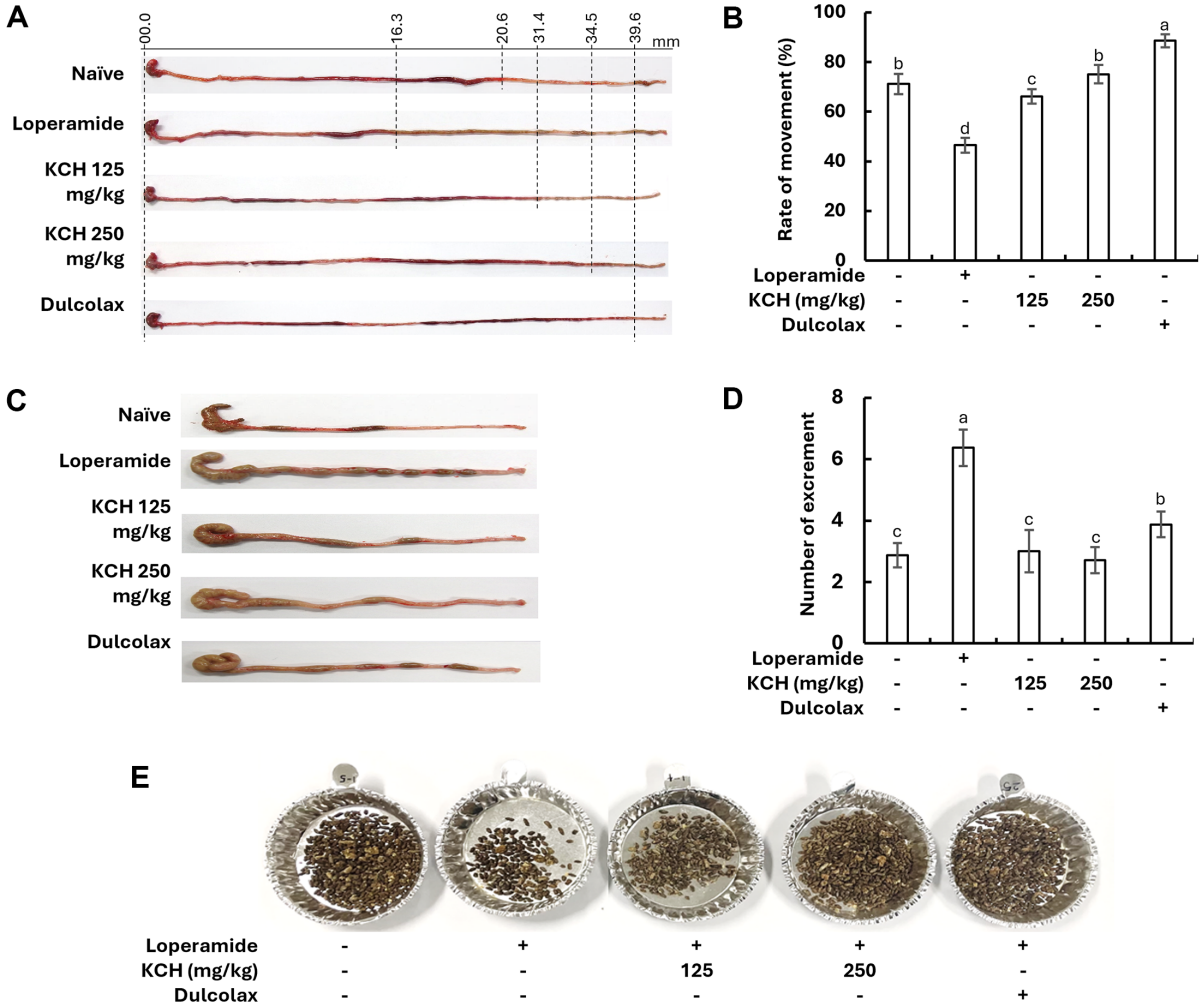
Effect of KCH on (A) the intestinal transit lengths, (B) the intestinal transit ratios, (C) the fecal impaction in the large intestine and the rectum, and (D) the number of fecal impacts in each experimental group in loperamide-induced constipated mice, and (E) the excrement changes on the third day after loperamide gavage. Data represent results from four independent experiments (*n* = 7 or 8) and are expressed as mean ± SE. Significantly different results are indicated by distinct lowercase letters (*p* < 0.05).

**Intestinal transit length and intestinal transit ratio.** Intestinal transit lengths of the KCH groups and Dulcolax group were increased compared to the constipation group as indicated in Fig. 7A. The small intestinal length increment observed in the KCH groups and Dulcolax group was unexpected. The intestinal transit ratios illustrated in the graph in Fig. 7B indicated increased levels in KCH treatment groups. The intestinal transit ratio of the constipation group was significantly lower than the control group as well as KCH and Dulcolax groups. Fecal impaction was increased in the constipation group, and it was reduced with the KCH treatments (Fig. 7C and 7D). Excrements collected on the last day (3^rd^ day of the loperamide gavage) in Fig. 7E indicated a reduction in the number of excrements in the loperamide group and dose-dependent increment in KCH groups in contrast to the control group.

**Fig. 8:**
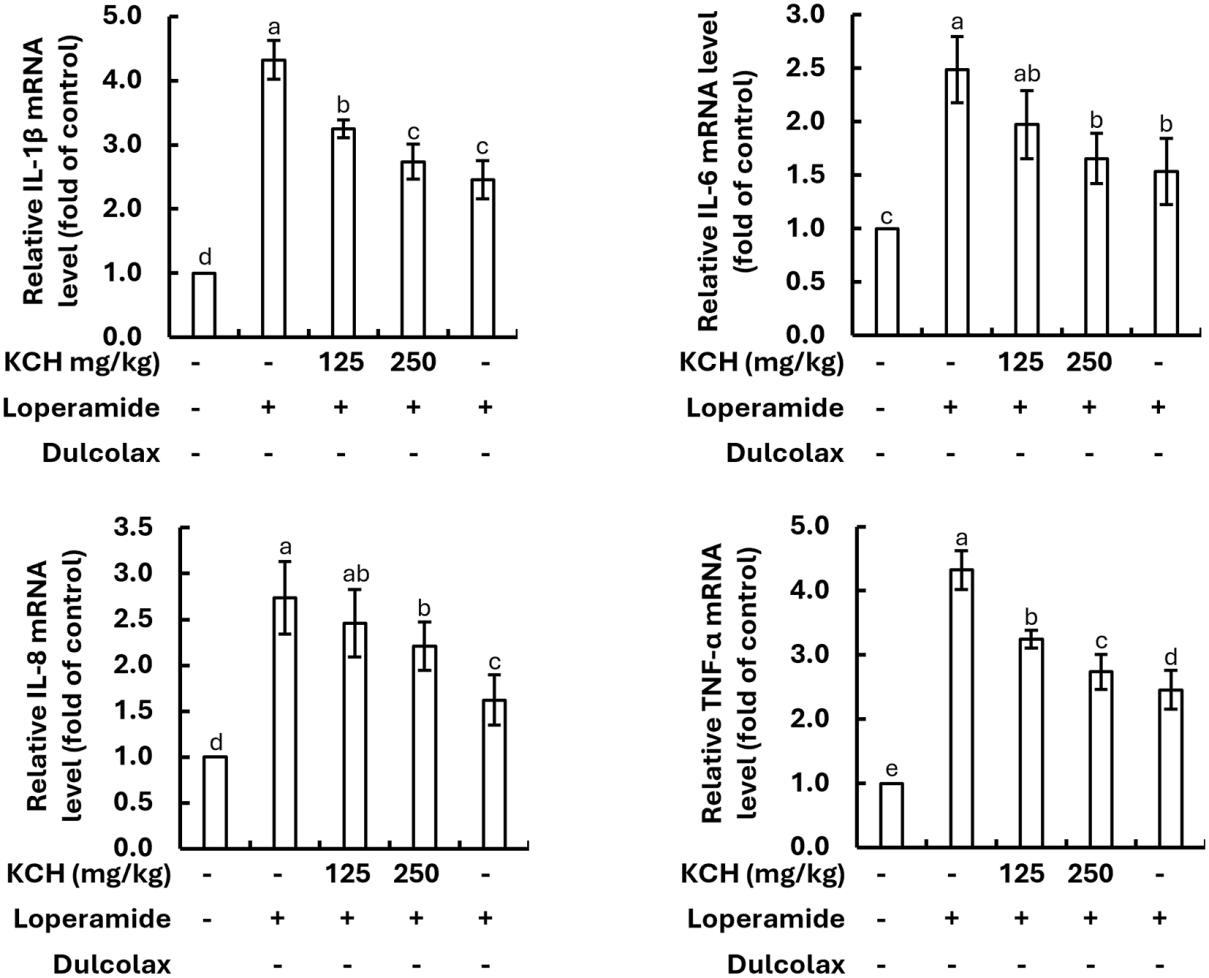
Effect of KCH on the mRNA expression of inflammatory cytokines in intestinal tissues of loperamideinduced constipated mice. Data are from three independent experiments (*n* = 3) and are expressed as mean ± SE. Significantly different results are denoted by distinct lowercase letters (*p* < 0.05).

**Effect on intestinal inflammatory cytokines.** The qRT-PCR analysis indicated the increased mRNA expression levels of IL-1β, IL-6, IL-8, and TNF-α in constipation-induced mice following loperamide stimulation. The oral administration of KCH significantly reduced the mRNA expression levels of IL-1β, IL-6, IL-8, and TNF-α in a dose-dependent manner (Fig. 8).

**Fig. 9:**
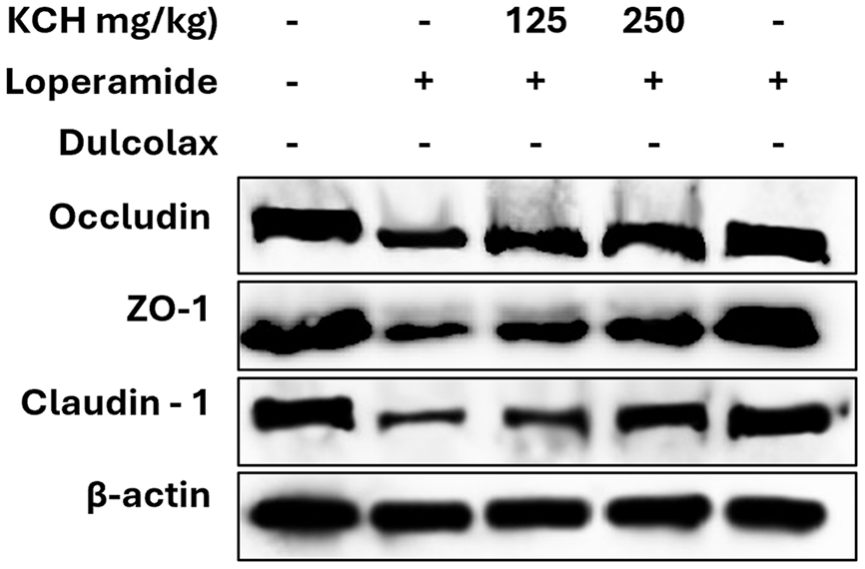
Effect of KCH on the intestinal barrier-related protein expression levels in intestine tissues of loperamide-induced constipated mice.

**Effect on intestinal barrier functions in constipated mice intestinal.** The protein expression of intestinal barrier-related molecules, including occludin, ZO-1, and claudin-1 was significantly enhanced by the KCH dose-dependently (Figs. 9 and S6).

## Discussion

IBD encompasses a group of chronic inflammatory conditions of the gastrointestinal tract, primarily Crohn's disease and ulcerative colitis [2]. It is characterized by prolonged inflammation in the small intestine and/or colon, which can cause damage to the digestive tract, leading to a range of symptoms such as abdominal pain, diarrhea/constipation, rectal bleeding, weight loss, and fatigue [29]. Even though the exact etiology is not completely understood, IBD involves a multifactorial etiology, including genetic susceptibility, immune system dysfunction, environmental influences, and changes in gut microbiota [30]. However, conventional treatments for these conditions and its symptoms have been indicated low effectiveness and serious side effects in clinical trials [31]. Therefore, the gap between IBD conditions and new therapeutic strategies must be filled to address the issue.

In the present study, we investigated the anti-inflammatory effect of KCH on LPS-stimulated Caco-2 cells. As results obtained from the cytotoxicity analysis on Caco-2 cells indicated, KCH concentrations below 125 μg/ml were used in further in vitro experiments. As reported, NO plays a crucial role in the inflammatory response in Caco-2 cells. Decreasing the NO production ameliorates the hyperpermeability of inflamed cells and is linked to the activation of inducible nitric oxide synthase (iNOS), which contributes to the inflammatory process [32, 33]. Moreover, iNOS and COX-2 together with the NO pathway have been proposed as a potential mechanism of action in tight junction (TJ) degradation [34]. Our findings confirmed the reduction in NO levels in LPS-stimulated Caco-2 cells with KCH doses. Results also showed that KCH inhibited iNOS, and COX-2 in a dose-dependent manner against LPS stimulation. While the precise mechanisms responsible remain largely unknown, pro-inflammatory cytokines are widely acknowledged for intestinal barrier dysfunction in IBD [31]. Interestingly, increased production of inflammatory cytokines in LPS-stimulated Caco-2 cells was dose-dependently decreased by the KCH treatment.

The overactivation of the MAPK/NF-κB pathway increases the expression of pro-inflammatory mediators. In addition, activation of the MAPK/NF-κB pathway is a key mechanism underlying the TJ disruption induced by the pro-inflammatory cytokines [35]. Results obtained from western blot analysis indicated the downregulation of the phosphorylation of p38, ERK, and JNK in MAPK signaling with KCH doses on LPS-stimulated Caco-2 cells. Furthermore, the KCH sample treatment downregulated the phosphorylation of NF-κB signaling molecules and suppressed the nuclear translocation of the p65 subunit in LPS-stimulated Caco-2 cells. It has been reported that overexpression of TNF-α downregulates the ZO-1 protein expression, disassembly of claudin-1, and induces the redistribution of this TJ protein away from the TJ via an NF-κB dependent mechanism in Caco-2 cells [34]. Furthermore, the dependent mechanism through IL-1β decreased the occludin expression in intestinal cells [36]. Outcomes of the western blot analysis indicated the upregulation of the ZO-1, claudin 1, and occludin molecules which were suppressed by LPS stimulation. Altogether, the above in vitro study confirmed the potential of KCH treatment as a preventive strategy for the management of IBD-like inflammatory disorders.

Functional foods with anti-inflammatory properties can help modulate immune responses, thereby reducing inflammation and promoting intestinal healing. It possesses laxative effects that can help regulate bowel movements, improve gut motility, and enhance overall gastrointestinal health [37]. Multifaceted functional foods ingredient incorporation into a strategy could offer a holistic approach to managing IBD and its associated symptoms, leading to improved patient outcomes. In the later part of the study, despite its anti-inflammatory effects, we investigated the multifaceted potential of KCH as a functional food material to manage constipation.

We observed that loperamide-induced constipation mice decreased fecal pellet number and weight due to the increased fecal evacuation time and low moisture content in the fecal. A previous report indicated that the inhibition of colonic peristalsis is responsible for the above observation [28]. The oral administration of the KCH to BAlb/c mice indicated an increase of stool movement in the small intestine which confirmed the effect in constipation management in the first place. Calculation on the rate of movement in individual groups indicated the dose-dependent anti-constipation effect of the KCH. As observed in the in vivo experiment, fecal impact was reduced with the KCH treatments. Excrements were collected on the last day. By observing it, we confirmed the reduction in the number of excrements in the loperamide group and the dose-dependent increment in KCH groups in contrast to the control group. Moreover, outcomes of the in vivo study observed that KCH suppressed colon inflammation by reducing the mRNA expression of pro-inflammatory cytokines (IL-1β, IL-6, IL-8, and TNF-α) in loperamide-induced constipated mice. Elevated levels of pro-inflammatory cytokines disrupt the expression and function of tight junction proteins, which increases intestinal permeability and contributes to the pathophysiology of constipation [38]. Interestingly, the oral administration of KCH increased the expression levels of tight junction proteins (occludin, ZO-1, and claudin-1) in mice colon tissues. Therefore, KCH indicated the potential to improve intestinal transit in loperamide-induced constipated mice. Collectively, an in vivo study confirmed the effectiveness of KCH in intestinal transit and alleviated constipation in loperamide-induced mice.

## Conclusion

Current research has exhibited the protective effects of KCH against inflammatory reactions and TJ protein destruction induced by LPS in Caco-2 cells. Results indicated the downregulation of pro-inflammatory mediators and inhibition of MAPK/NF-κB activation in Caco-2 cells suggesting the potential usability of KCH against IBD-like conditions. Moreover, this study demonstrated that KCH enhanced intestinal transit and alleviated constipation in loperamide-induced BAlb/c mice while reducing the mRNA expression levels of pro-inflammatory cytokines and increasing the expression levels of tight junction proteins in intestinal tissues collected from constipated mice. These findings indicate the potential of KCH as a functional food ingredient for suppressing inflammation-related disorders, constipation, and its pathophysiology.

## Supplemental Materials

Supplementary data for this paper are available on-line only at http://jmb.or.kr.



## Figures and Tables

**Table 1:** Specifications of in vivo experimental groups and treatments in each group.

Experimental group	Number of mice	Acclimatization for 7 days	Treatments		
			7-21 day	22-24 day	25^th^ day
Control	8	Yes	Normal saline solution 200 μl	Normal saline solution 200 μl	6 % Carmine^b^
Constipation	8	Yes	Normal saline solution 200 μl	Loperamide 10 mg/kg^a^	6 % Carmine
KCH 125	7	Yes	KCH 125 mg/kg^a^	Loperamide 10 mg/kg^a^	6 % Carmine
KCH 250	7	Yes	KCH 250 mg/kg^a^	Loperamide 10 mg/kg^a^	6 % Carmine
Positive control	8	Yes	Dulcolax 5.5 mg/kg^a^	Loperamide 10 mg/kg^a^	6 % Carmine

^a^Concentrations were calculated according to the mg of samples for kg of body weight.

^b^6% Carmine was prepared by mixing 50 mL of 0.5% carboxymethyl cellulose and 3 g of carmine.

**Table 2:** Proximate composition of KCH.

Yield (%) ^a^	Proximate composition (%) ^a^		
	Carbohydrate	Protein	Total polyphenol
27.61 ± 1.89	11.79 ± 0.24	8.12 ± 0.21	2.80 ± 0.10

^a^Yield and proximate composition were indicated as % in dry basis.

All experiments were performed in triplicate (*n* = 3) to determine the repeatability and results were expressed as mean ± standard error of mean.
