# Supplementary material for: Kjellmaniella crassifolia Reduces Lipopolysaccharide-Induced Inflammation in Caco-2 Cells and Ameliorates Loperamide-Induced Constipation in Mice
Source: J Microbiol Biotechnol. 2024 Oct 1;34(12):2565–75. doi: 10.4014/jmb.2407.07036 (PMC11729519; doi:10.4014/jmb.2407.07036)
Supplement: Supplementary file 1 [file jmb-34-12-2565-supple.pdf]

Supplementary material

Kjellmaniella crassifolia Reduces Lipopolysaccharide-induced Inflammation in Caco-2 Cells and Ameliorates Loperamide-induced Constipation in Mice

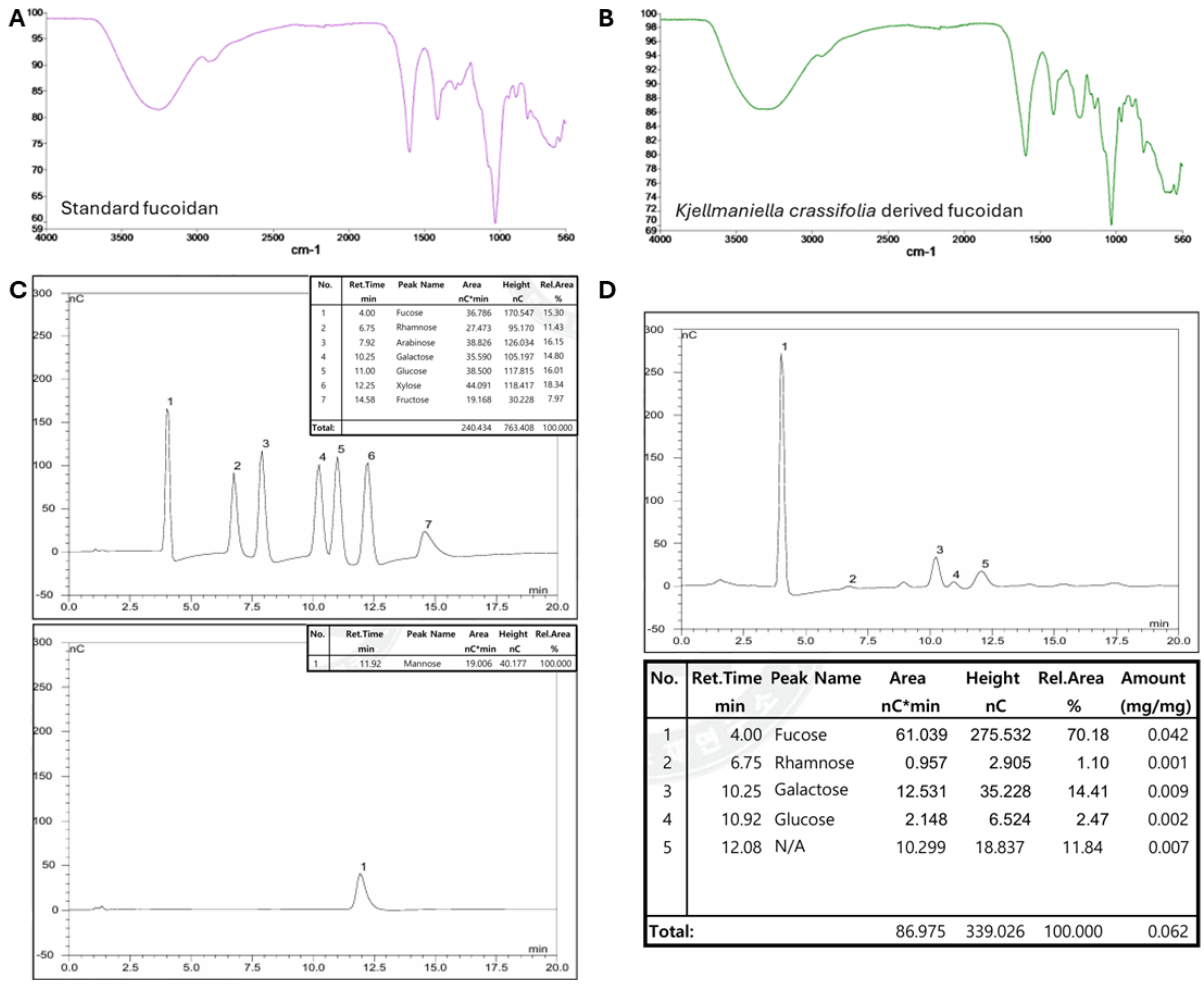

No.	Ret.Time min	Peak Name	Area nC*min	Height nC	Rel.Area %
1	4.00	Fucose	36.786	170.547	15.30
2	6.75	Rhamnose	27.473	95.170	11.43
3	7.92	Arabinose	38.826	126.034	16.15
4	10.25	Galactose	35.590	105.197	14.80
5	11.00	Glucose	38.500	117.815	16.01
6	12.25	Xylose	44.091	118.417	18.34
7	14.58	Fructose	19.168	30.228	7.97
Total:			240.434	763.408	100.000

No.	Ret.Time min	Peak Name	Area nC*min	Height nC	Rel.Area %
1	11.92	Mannose	19.006	40.177	100.000

No.	Ret.Time min	Peak Name	Area nC*min	Height nC	Rel.Area %	Amount (mg/mg)
1	4.00	Fucose	61.039	275.532	70.18	0.042
2	6.75	Rhamnose	0.957	2.905	1.10	0.001
3	10.25	Galactose	12.531	35.228	14.41	0.009
4	10.92	Glucose	2.148	6.524	2.47	0.002
5	12.08	N/A	10.299	18.837	11.84	0.007
Total:			86.975	339.026	100.000	0.062

Fig. S1. FTIR spectrum of (A) Commercially available standard fucoidan obtained from Sigma, (B) *Kjellmaniella crassifolia* fucoidan and HPAEC-PAD spectrum of and (C) Commercially available standard mono-sugar mixture and (D) Kjellmaniella crassifolia fucoidan.

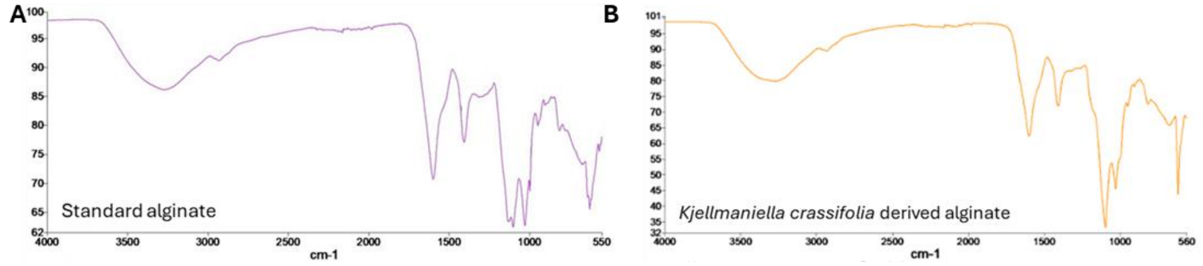

Fig. S2. FTIR spectrum of (A) Commercially available standard alginate obtained from Sigma, (B) *Kjellmaniella crassifolia* alginate.

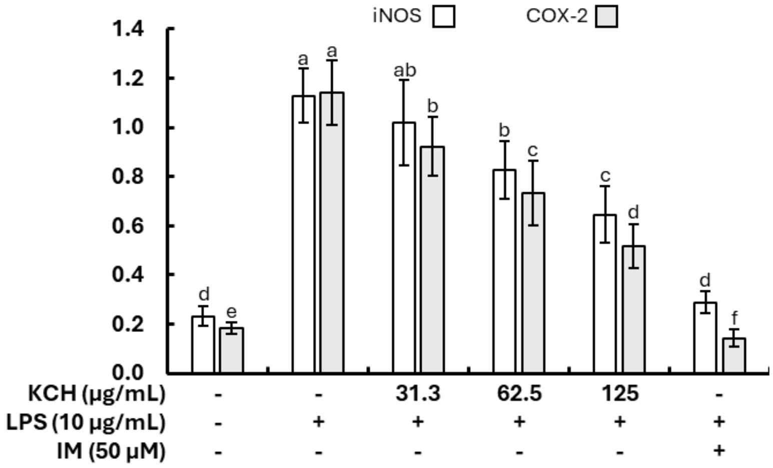

Fig. S3. Relative folds (molecule/beta actin) of iNOS and COX-2 in LPS-stimulated Caco-2 cells.

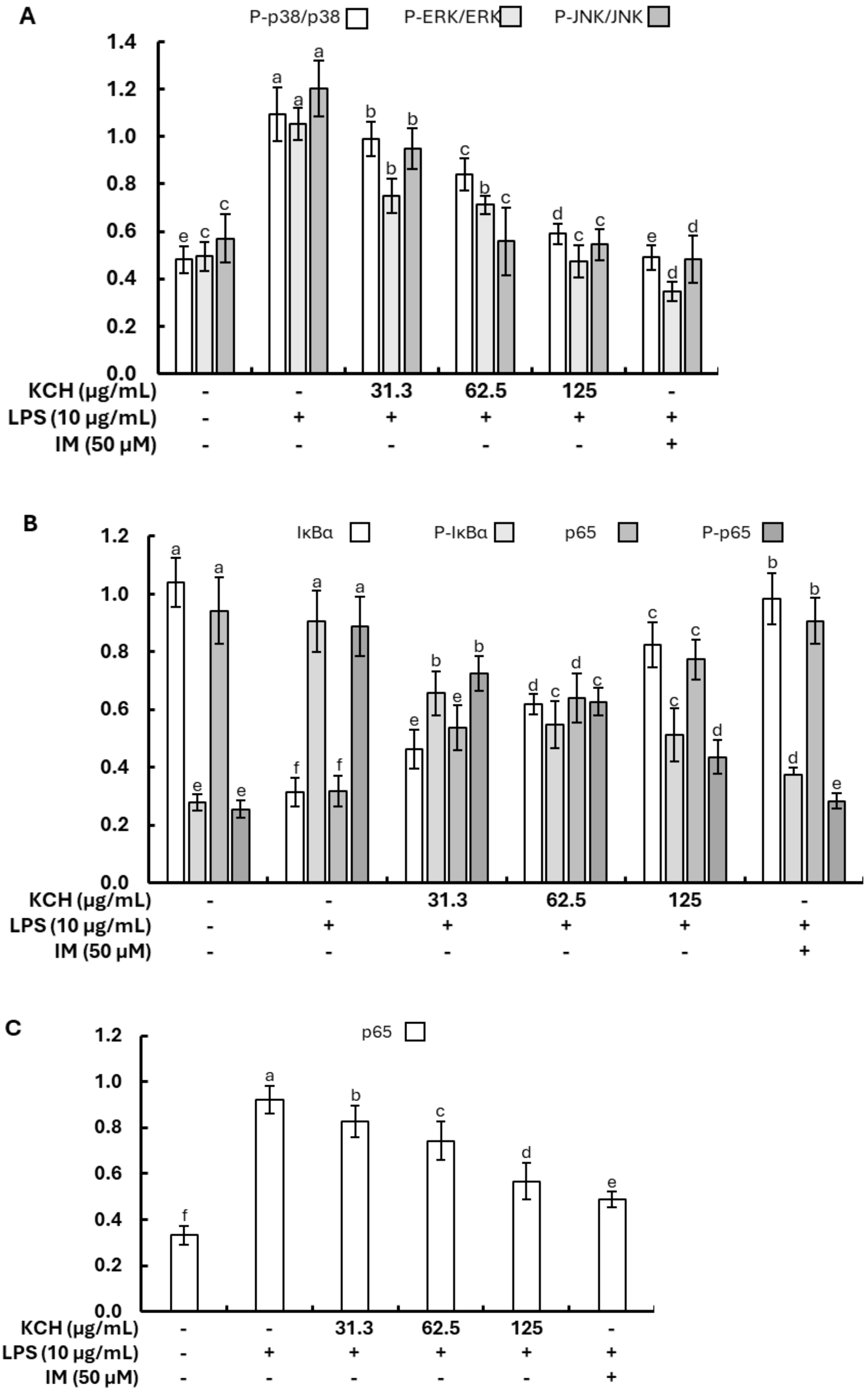

Fig. S4. (A) Folds of MAPK singnaling molecules, (B) Relatine folds (molecule/beta actin) of cytosolic NFkB signalin molecules, and (C) Relatine folds (p65/Lamin b) of nucleic NFkB p65 signalin molecule.

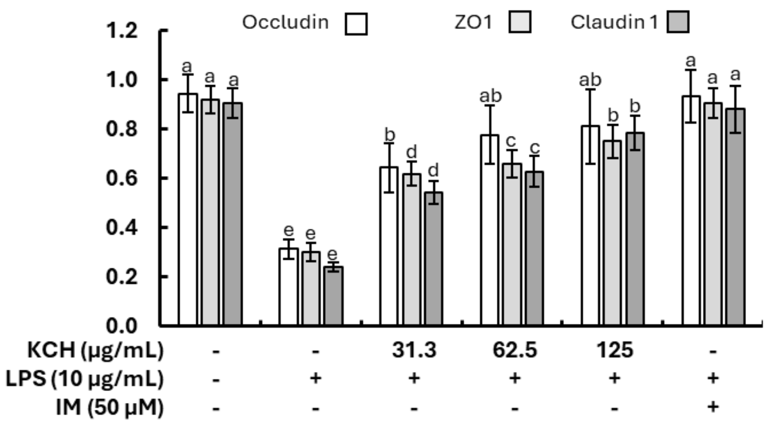

Fig. S5. Relatine folds (molecule/beta actin) of tight junction proteins in LPS-stimulated Caco-2 cells.

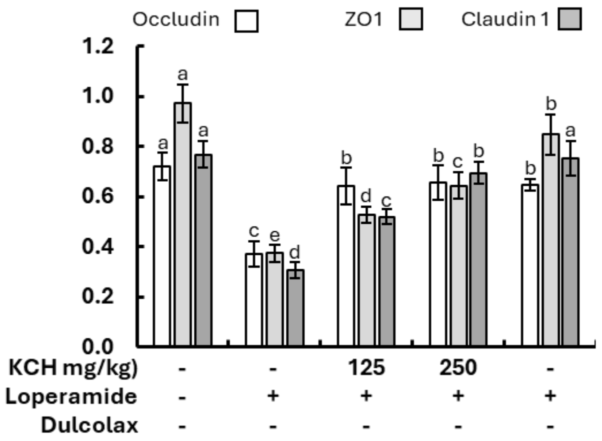

Fig. S6. Relatine folds (molecule/beta actin) of tight junction proteins in intestinal tissues of Loperamide induced constipated mice.
